# Inflammation, Nitro-Oxidative Stress, Impaired Autophagy, and Insulin Resistance as a Mechanistic Convergence Between Arterial Stiffness and Alzheimer’s Disease

**DOI:** 10.3389/fmolb.2021.651215

**Published:** 2021-03-29

**Authors:** Jhana O. Hendrickx, Wim Martinet, Debby Van Dam, Guido R. Y. De Meyer

**Affiliations:** ^1^Laboratory of Physiopharmacology, Faculty of Pharmaceutical, Biomedical and Veterinary Sciences, University of Antwerp, Antwerp, Belgium; ^2^Laboratory of Neurochemistry and Behavior, Institute Born-Bunge, Department of Biomedical Sciences, University of Antwerp, Antwerp, Belgium; ^3^Department of Neurology and Alzheimer Research Center, University of Groningen and University Medical Center Groningen, Groningen, Netherlands

**Keywords:** inflammaging, metabolism, nitro-oxidative stress, autophagy, neurodegeneration, arterial stiffness

## Abstract

The average age of the world’s elderly population is steadily increasing. This unprecedented rise in the aged world population will increase the prevalence of age-related disorders such as cardiovascular disease (CVD) and neurodegeneration. In recent years, there has been an increased interest in the potential interplay between CVDs and neurodegenerative syndromes, as several vascular risk factors have been associated with Alzheimer’s disease (AD). Along these lines, arterial stiffness is an independent risk factor for both CVD and AD. In this review, we discuss several inflammaging-related disease mechanisms including acute tissue-specific inflammation, nitro-oxidative stress, impaired autophagy, and insulin resistance which may contribute to the proposed synergism between arterial stiffness and AD.

## Introduction

The average age of the world population is steadily increasing with approximately 1 billion people in 2019 that were aged 65 or older. By 2030, this number is estimated to increase to 1.4 billion, reaching 2.1 billion by 2050 (WHO, 2018). This unprecedented rise in the aged world population will increase the prevalence of age-related disorders such as cardiovascular disease (CVD) and dementia. Globally, CVD is the number one cause of death with an estimated mortality of 17.9 million people in 2016 (WHO, 2017). That same year, 43.8 million people worldwide suffered from dementia, which was the fifth leading cause of global death with 2.4 million people dying from this disease (GBD 2016 Neurology Collaborators, 2019). Historically, CVD and Alzheimer’s disease (AD) were considered to be separate entities based on clinical classification criteria. However, increasing epidemiological studies report an independent convergence between both diseases, suggesting a mechanistic overlap. In this review, we discuss chronic low-grade inflammation as the mechanistic convergence between CVD and AD given its major contribution in both pathologies.

### Alzheimer’s Disease

For many centuries, dementia has been described in ancient texts (e.g., “*Be kind to your father, even if his mind fails him.*” – Old Testament: Sirach 3:12). In itself, dementia comprises a plethora of neurological syndromes with the overall clinical symptom of a progressive cognitive disturbance affecting the patient’s independent functionality in everyday life. These cognitive disturbances are commonly accompanied by changes in behavior, mood, and personality (Tariot et al., 1995; Mega et al., 1996). In the past, a distinction was often made between primary degenerative dementias, such as dementia with Lewy bodies, frontotemporal dementia, and AD. However, since The Lancet International Conference on Dementias in 1966 (Eastwood et al., 1996), the genesis of dementia syndromes was elucidated by reassessing this simplistic distinction as a complex synergism of (epi)genetic predisposition, lifestyle factors, psychosomatic, and neuropathological changes. Now it is clear that age is the overarching common factor, given the singular or additive effects of pathologies on dementia. Current diagnostic criteria for dementia incorporate advances in scientific knowledge and technological progression in the detection and understanding of dementia and related disorders associated with cognitive impairment. The most recent edition of the Diagnostic and Statistical Manual of Mental Disorders (DSM-5) renamed dementia to major neurocognitive disorder (NCD), although the label dementia is still of course interchangeably used with major NCD. DSM-5 also distinguishes between major NCD and mild NCD, which is perhaps better known as mild cognitive impairment (MCI) or prodromal dementia (Vahia, 2013). Specifically, for AD, as the prototype of cortical dementia, the National Institute on Aging and Alzheimer’s Association described separate diagnostic criteria and related recommendations for the preclinical, MCI, and dementia stages of AD, for the first time also considering *in vivo* biomarkers, based on neuroimaging and the determination of disease-linked compounds in bodily fluids (Jack et al., 2018).

The conceptualization of AD dates from 1907 when Alois Alzheimer reported the presence of neuropathological amyloid plaques and neurofibrillary tangles (NTFs) after the *post-mortem* brain analysis of 55-year-old Auguste Deter (Maurer et al., 1997). Over the past decades, molecular biology and genetic research have led to an improved understanding of these two key neuropathological lesions. Additional lesions include cerebral amyloid angiopathy (CAA), dystrophic neurites, neuropil threads, astrogliosis, and microglial activation (Serrano-Pozo et al., 2011). As the most prominent form of dementia, AD accounts for an estimated 60–80% of dementia patients aged 65 or older (Alzheimer’s Association, 2015). Most patients present with an anterograde amnestic syndrome besides retention of social graces due to mesial temporal lobe atrophy (McKhann et al., 2011; Orgeta et al., 2015). That is why the World Health Organization recognizes AD as a global public health priority. The vast majority of late-onset AD cases occur on a sporadic basis, driven by a complex combination of genetics and environmental factors of which 70% is thought to be attributable to genetics. While aging is still the biggest risk factor of late-onset AD, genetic risk factors have also been identified. The most common genetic risk factor comprises polymorphisms in one of the three common alleles (ε2, ε3, and ε4) of the *APOE* gene of which mutations in the ε4 allele carry to the most increased risk (Hardy, 1995; Verghese et al., 2011; AlzGene, 2020a). Because this *APOE* genotype is linked to cholesterol transport dynamics, it has been suggested that it is a vascular risk factor of late-onset AD (Kalaria et al., 2012). Besides polymorphisms in the *APOE* gene, several novel risk genes have been suggested which are linked to cholesterol metabolism (*ABCA7*), inflammation (*TREM2*), amyloid beta (Aβ) clearance (*CLU*), the immune system (*CR1*) among others (AlzGene, 2020b). The less common early-onset AD cases are mostly attributable to inheritable mutations in the *APP*, *MAPT*, *PSEN1*, and *PSEN2* gene (Haass and De Strooper, 1999; Bateman et al., 2011; AlzForum, 2020). Recently, age-dependent differences in mRNA expression levels of the aforementioned late-onset AD genetic risk factor, *TREM2*, have been found in early-onset AD cases. This finding indicates that the *TREM2* gene might be a non-hereditary risk factor for early-onset AD (Guven et al., 2020). Although advances in medical technology in the past have led to longer survival, there is still a lack of disease-modifying treatments.

### Amyloid Plaques

One of the major pathological hallmarks of both early- and late-onset AD is the extracellular accumulation of abnormally folded Aβ peptides in amyloid plaques. Aβ peptides are 36–42 amino acids long proteolytic fragments derived from the transmembrane and extracellular domains of the amyloid precursor protein (APP), which localizes genetically on chromosome 21. APP can be processed via either the amyloidogenic pathway, thereby releasing Aβ peptides after cleavage at both the γ-secretase and β-amyloid cleavage enzyme (BACE) sites, or via the non-amyloidogenic pathway in which α-secretase cleaves within the Aβ sequence to release a neuroprotective sAPPα fragment (Nunan and Small, 2000). Monomeric Aβ peptides tend to aggregate as oligomers, protofibrils, and mature amyloid fibrils. Amyloid plaques mainly constitute of Aβ1–40 and Aβ1–42 peptides with the latter type being most prone to aggregation into amyloid plaques (Hardy, 1997). Mutation clusters around the three secretase cleaving sites of APP are known to cause familial AD, with mutations leading to an increased production of total Aβ or an increased Aβ1–42/Aβ1–40 ratio (AlzForum, 2020).

Several types of plaques have been described ranging from diffuse plaques, being amorphous amyloid deposits, over dense-core plaques with fibrillar amyloid deposits that are typically surrounded by dystrophic neurites (neuritic plaques), reactive astrocytes and activated microglial cells, and culminating into burnt-out plaques (Serrano-Pozo et al., 2011). Amyloid plaques primarily accumulate in the neocortex, often involving all six neocortical layers, continuing to the allocortex and finally also progressing to subcortical regions (Arnold et al., 1991; Thal et al., 2002). Various scoring systems have been implemented to stage amyloid plaque burden and spreading. Initially, three stages (A through C) were distinguished (Braak and Braak, 1991). Thal et al. (2002) proposed five stages (1 through 5) to describe the spatiotemporal progression of amyloid pathology.

### Cerebral Amyloid Angiopathy

Apart from the parenchymal accumulation of Aβ, approximately 80–90% of AD patients also exhibit Aβ deposition in cerebrovascular vessels, referred to as CAA (Yamada et al., 1987; Yamada, 2002). CAA is mainly observed in leptomeningeal and cortical vessels (Yamada et al., 1987). In contrast to amyloid plaques, cerebrovascular Aβ segments are mainly 40 amino acids in length (Prelli et al., 1988; Suzuki et al., 1994). After neuronal release, Aβ1–42 aggregates in amyloidogenic plaques in brain parenchyma, while Aβ1–40 is transported via interstitial fluid drainage to the cerebrovasculature for clearance. In this process, Aβ1–40 can aggregate on and deposit within cerebrovascular basement membranes (Weller et al., 1998). Affected vessels can show secondary vasculopathic changes, such as loss of smooth muscle cells, wall thickening, fibrinoid necrosis, formation of micro aneurysms, and deposition of perivascular blood breakdown products (Love et al., 2015).

### Neurofibrillary Tangles

Neurofibrillary tangles (NFTs) are another neuropathological hallmark of the AD brain. The major components of the intracellular NFTs are paired helical filaments of hyperphosphorylated microtubule-associated protein tau (MAPT) (Serrano-Pozo et al., 2011). MAPT forms a crucial neuronal component via the assembly and stabilization of the microtubule cytoskeleton which is essential for axonal transport (Paglini et al., 2000). Cytoskeleton disassembly impairing axonal transport and aggregation of hyperphosphorylated tau into fibrils are early events of AD pathogenesis and significantly impact neuronal functioning (Nagy et al., 1995). The spatiotemporal distribution of tauopathy follows the reversed pattern to amyloid burden in AD. Tau pathology in AD develops progressively in synaptically connected brain regions presumably based on transcellular propagation of tau aggregates (Furman et al., 2017). NFTs arise earliest in the entorhinal cortex and hippocampus located in the medial temporal lobe and further spread to the associative isocortex, thereby relatively exempting primary sensory, motor, and visual areas. NFTs exhibit a characteristic distribution pattern permitting the differentiation of six Braak stages (I through VI) (Braak and Braak, 1991).

Currently, AD neuropathology staging is predominantly based on Montine’s ABC scoring system that combines Aβ deposit staging as described by Thal et al. (2002) (A score), Braak NFT staging (B score) (Braak and Braak, 1991), and finally neuritic plaque scoring (C score) (Montine et al., 2012). Neuritic plaques are characterized by a central core of Aβ aggregates surrounded by a corona composed of degenerating neurons with dystrophic neurites and reactive astroglia and microglia.

## Arterial Stiffness as a Risk Factor of Alzheimer’s Disease

In recent years, there has been an increased interest in the potential contribution of vascular diseases in the AD pathogenesis because several vascular risk factors have been associated with this dementia syndrome, e.g., hypertension, metabolic syndrome, hypercholesterolemia, atherosclerosis, hyperlipidemia, certain forms of coronary disease, and more recently arterial stiffness. Among these vascular risk factors of AD, hypertension appeared to be the strongest (Kennelly et al., 2009). Most clinical studies demonstrate that mid-life hypertension predisposes to the AD dementia syndrome in late life (Skoog et al., 1996; Kivipelto et al., 2001) as long-standing increments in blood pressure and blood pressure variations induce anatomical and functional alterations in the brain such as white matter changes, cerebral hypoperfusion due to atherosclerosis or the disruption of vasoregulatory functions (Kalaria, 2010; Lattanzi et al., 2014, 2015). Moreover, class-specific and dose-dependent antihypertensive therapies decrease the incidence of MCI and dementia (Takeda et al., 2009) and reduce the AD pathogenesis (Hoffman et al., 2009), making blood pressure an interesting therapeutic target. However, a global clinical study, including 18,017 hypertensive patients, concluded that merely 32% of patients treated with anti-hypertensives resulted in systolic blood pressure (SBP) control (Thoenes et al., 2010). Later, the REASON study (Protogerou et al., 2009) was able to explain this poor clinical outcome as they found a positive correlation between SBP and arterial stiffness, a strong independent predictor of CVD.

Already in the 17th century the concept of arterial stiffness was exemplified by the physician Thomas Sydenham (1624–1688) with his famous dictum “*a man is as old as his arteries*” This phrase is still valid because recent aging theories suggest that changes in vascular anatomy are an important determinant of an organism’s fate (Learoyd and Taylor, 1966; Bailey, 2001; Lakatta and Lévy, 2003; Greenwald, 2007). With every heartbeat, nutrient-rich, oxygenated blood is ejected from the heart’s ventricles into the arterial tree. The cyclic nature of the propagation and reflection of blood pulse waves not only define the mechanical force experienced by the vasculature but also the load imposed on each ventricle (Minor, 1982). This hemodynamic conduction behavior is maintained in mammals, where the mean pulse pressure at the base of the brain is remarkably similar between mice, dogs, humans, and even giraffes, whose hearts have to work a little harder to ensure optimal peripheral perfusion (Noordergraaf et al., 1979; Kass et al., 1988; Beyar et al., 1989). Overall, this cardiovascular phenomenon underscores an often ignored but profound characteristic of the cardiovascular circulation, namely that it is pulsatile (Kass et al., 1988).

In addition, the cardiovascular system ensures that the high-pressure blood flow, generated by the intermittent pumping of the heart to the periphery, is captured to maintain a stable perfusion in vulnerable vascular beds with high and low resistance (Vlachopoulos et al., 2011). This dampening effect is accurately assessed in the Windkessel model as the ability of large arteries to immediately adapt to changing ejected blood volumes and to store the excess stroke volume during systole and to drain it during diastole (Chau et al., 1982; Safar et al., 2003; Ben-Shlomo et al., 2014). Under physiological conditions, the stiffness of the arteries gradually increases from the heart to the periphery, which is partly due to the proximodistal narrowing of the arterial diameter. This stiffness gradient causes an impedance mismatch that induces partial wave reflections to reduce the transmission of pulsatile energy and to protect the microcirculation (London and Pannier, 2010). The concept of wave reflections is best understood by differentiating between velocity and pulse waves in the ascending aorta. Where the velocity wave is a single spurt from the heart that drops to zero at the incisura during the entire diastolic cycle, the pulse wave is reflected in two localized peaks, the first of which corresponds to the flow peak and the second to the summation of the reflection in that specific part of the body (London and Pannier, 2010; O’Rourke et al., 2014).

Whereas the Windkessel model is a non-propagative model of arterial stiffness, a more appropriate approach was introduced by Adriaan Isebree Moens (Dow, 1940) and Diederik Korteweg (Tijsseling and Anderson, 2012) with the Moens–Korteweg equation that describes both propagation and velocity. Given that pulse waves travel faster in stiffer arteries, the verification of pulse wave velocity (PWV) was suggested as a more reliable measurement of arterial stiffness (Safar and London, 1987). Recently, the Scandinavian physiologist Bjorn Folkow described how aging can distort the regulation of arterial function where progressive arterial stiffness causes increased arterial impedance and pulsatile pressure in the arterial tree (Folkow and Svanborg, 1993). As arterial tissue loses its elasticity with age, increased pulsatile strain damages the most vulnerable microcirculations (O’Rourke and Safar, 2005) such as the cerebrovasculature, leading to severe lesions and end-organ failure, e.g., cognitive decline and cerebral bleeding (O’Rourke and Hashimoto, 2007).

Under physiological conditions, arterial stiffness increases progressively from the heart to the periphery since the proximal aorta is more distensible than the distal tract. Together with the proximo-distal tapering of the arterial diameter, the stiffness gradient determines an impedance mismatch that induces reflection of the pulse wave. Partial wave reflections reduce the transmission of pulsatile energy to the periphery and, hence, protect the microcirculation. When proximal aortic stiffness increases and the physiological gradient is reduced or reverted, the pulse propagation cannot be adequately dampened, is transmitted to smaller arteries, and impinges on the microcirculation (Noordergraaf et al., 1979). In addition, less reflected waves return to the central aorta and increase systolic BP and pulse pressure by superimposing on the incident pulse waves (Tariot et al., 1995).

## Link Between Arterial Stiffness and Alzheimer’s Disease

**FIGURE 1:**
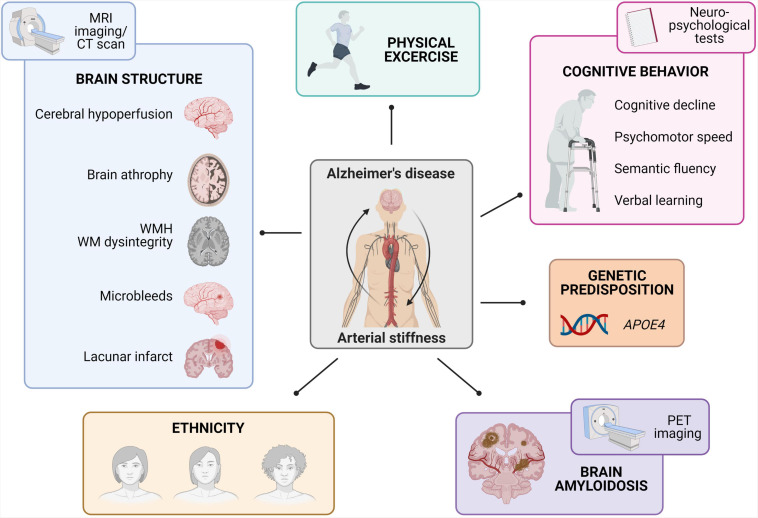
Summary of epidemiological findings linking arterial stiffness to Alzheimer’s disease. The observed links between arterial stiffness and Alzheimer’s disease comprise brain structure changes (as studied via MRI-based imaging techniques), physical fitness, cognitive behavior (as studied with neuropsychological tests), *APOE4* genetic predisposition, brain amyloidosis (as studied via Aβ-PET imaging), and differences in ethnicity (MRI, magnetic resonance imaging; MWH, white-matter-hyperintensities; WM, white matter; PET, positron emission tomography).

In essence, arterial stiffness relates the pulsatility of the heart to the brain. Given its extensive microvasculature, the brain is not only a low-resistance but also a high-flow organ that is continuously exposed to cardiac pulsatile pressures and mechanical forces (O’Rourke and Safar, 2005). There is a consensus that heightened PWV is linked to a faster cognitive decline, changes in psychomotor speed and difficulties in semantic fluency and verbal learning despite discrepancies between studies in terms of study design (longitudinal vs. cross-sectional), cognitive screening, target populations, and their heterogeneity (Rabkin, 2012; Hughes et al., 2015; van Sloten et al., 2015; Iulita et al., 2018). In this context, the ARIC-NS study concluded that higher arterial stiffness and pulsatility were associated with MCI and dementia mainly in Caucasian participants. Also the recent ASCEND study reported the influence of ethnicity on peripheral vascular health in a healthy middle-aged cohort at risk for AD (Meyer et al., 2017). The study concluded that African Americans had worse peripheral vascular health and cognition compared to non-Hispanic White Americans (Kumar et al., 2020). Furthermore, physical excercise has been reported as a strong predictor of both arterial stiffness and cognition in the elderly. In general, a greater exercise engagement predicted better cognition in those with lower fitness, while arterial stiffness measurements were on their turn indicative of an individual’s physical fitness and cognitive status (Asamoah et al., 2013; Nascimento et al., 2019; Pereira et al., 2019; Kennedy et al., 2020; Mason et al., 2020). Additionally, genetic predisposition linked to the *APOE4* gene was recently proposed to act synergistically with arterial stiffness to predict cognitive impairment in non-demented elderly (Rodrigue et al., 2013; Cambronero et al., 2018; Rivera-Rivera et al., 2020; Smirnov et al., 2020; Figure 1).

Evaluation of altered brain structure due to arterial stiffness has been extensively investigated through cross-sectional and longitudinal epidemiological studies using MRI images and/or CT scans. One of the most specific recurring findings was the correlation between increased PWV measurements and a higher incidence of greater white matter hyperintensities and white matter dysintegrity (Henskens et al., 2008a,b; Coutinho et al., 2011; Mitchell et al., 2011; Poels et al., 2012; Tsao et al., 2013; Pase et al., 2016; Arai et al., 2018; Suri et al., 2020). Other brain screening programs also reported that PWV values were related to brain atrophy (Tsao et al., 2013; Pase et al., 2016). On a cerebrovascular level, heightened PWV values correlated to cerebral infarcts (Matsumoto et al., 2007; Henskens et al., 2008b; Tsao et al., 2013), microbleeds (Henskens et al., 2008a,b) and the presence of cerebral hypoperfusion (Muller et al., 2007; Watson et al., 2011; Jefferson et al., 2018; Suri et al., 2020). Other research groups applied Aβ-PET imaging in combination with PWV measurements in large human cohorts in order to investigate the convergence between arterial stiffness and AD. Overall, arterial stiffness was independently associated with a greater brain Aβ burden and the presence of CAA (Hughes et al., 2013, 2014, 2018; Moon et al., 2019; Pasha et al., 2020). Taken together, imaging-based cross-sectional and longitudinal studies in both symptomatic and asymptomatic subjects emphasize the association between increased PWV measures and cerebral damage in terms of cerebrovascular damage, neurodegeneration, an increased Aβ burden and CAA (Figure 1).

**FIGURE 2:**
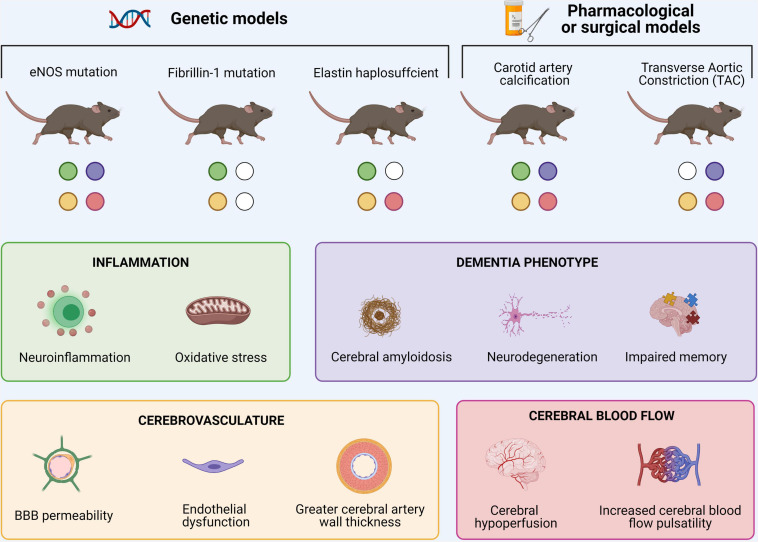
Overview of murine models of large arterial stiffness and associated cerebral findings. Common symptoms in these rodents comprise increased cerebral neuroinflammation, changes in cerebrovascular structure and/or cerebral blood flow and typical dementia-related features.

Although overwhelming evidence highlights the association between arterial stiffness and AD in (a)symptomatic human subjects, these studies are limited by correlative evidence. Animal models of large artery stiffness on the other hand, allow researchers to better investigate the causative mechanisms linking both diseases, though each with their own strengths and limitations. Examples include murine models with a partial or complete *eNOS* deletion (Austin et al., 2013; Tan et al., 2015; Li et al., 2018; Austin and Katusic, 2020), displaying heterozygous fibrillin 1 (Leloup et al., 2018) and elastin (Hughes et al., 2014; Said et al., 2018), carotid artery calcification (Muhire et al., 2019; Neuner et al., 2019), and transverse aortic constriction (Demer and Tintut, 2008; Steppan et al., 2012; Cooper and Mitchell, 2016). Common symptoms in these rodents comprise an increased (neuro)inflammatory state, changes in cerebrovascular structure and/or blood flow and a dementia phenotype alongside altered arterial stiffening and blood pressure features (Figure 2). Although proof-of-concept evidence has been established, knowledge gaps of the related causative mechanisms of arterial stiffness on AD remain. Identifying commonalities between these available models and their continuous characterization is therefore crucial. An additional hampering factor is the fact that rodents do not naturally accumulate Aβ. Therefore, the combination of large artery stiffness and transgenic AD models is needed. From a future perspective, more attention should be paid to possible effects of gender differences and influences of genetic background (Qosa and Kaddoumi, 2016; Neuner et al., 2019). Once the mechanisms underlying the convergence between arterial stiffness and AD are solidified, more precise interventions can be developed to delay the onset and/or progression of the AD pathogenesis.

## Causes of Arterial Stiffness

**FIGURE 3:**
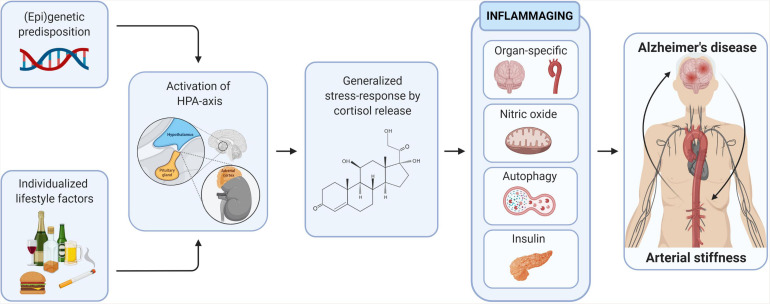
Inflammaging as the mechanistic convergence between arterial stiffness and Alzheimer’s disease. The long-term causal effect of (epi)genetic predisposition and individualized lifestyle factors and consequent generalized stress-response on several inflammaging-related drives the mechanistic convergence between arterial stiffness and Alzheimer’s disease.

Epidemiological research clearly indicates that chronological aging is the main determinant of arterial stiffness (Ishida et al., 2018; Namba et al., 2019). Aging is a ubiquitous complex phenomenon caused by a subtle blend of (epi)genetic alterations and individualized lifestyle factors (LaRocca et al., 2017; Morris et al., 2019; Wahl et al., 2019; Zhang et al., 2020b). Prolonged and cumulative exposure to these stimuli dysregulates and hyperactivates one of the body’s major stress system, being the hypothalamus-pituitary-adrenal (HPA)-axis, causing a generalized stress response by the release of stress hormones such as cortisol (Starr et al., 2019). A dysregulated HPA-axis negatively impacts overall health and activates the body’s immuno-inflammatory system (Tapp et al., 2019; Ahmad et al., 2020). Rather than being considered as an individual pathophysiology that comprises a simple “cause or consequence” connection, we propose that stress-driven, age-related perturbation of a chronic low-grade pro-inflammatory status, coined as inflammaging, (Franceschi et al., 2007; Chadwick et al., 2012) underlies arterial stiffness. Non-resolved inflammaging determines lifespan and the speed of aging, and is therefore highly associated with aging-related diseases including AD (Giunta et al., 2008), as well as with arterial stiffness (Fukami et al., 2020), insulin resistance (IR) (Itariu and Stulnig, 2014), nitro-oxidative inflammation (Miquel, 2009), and an impaired autophagic machinery (Salminen et al., 2012). The other way around, the deleterious effect of elevated glucocorticoid levels has been demonstrated in inflammaging-associated diseases such as AD (Khalsa, 2015; Justice, 2018), arterial stiffness (Vlachopoulos et al., 2009), acute inflammation (Guerrero, 2017), impaired autophagy (Ma et al., 2019), nitro-oxidative stress (Bernatova et al., 2018), and IR (Burke et al., 2017). Aside from the overwhelming linkage between arterial stiffness and AD, recent evidence implies that arterial stiffness in itself is also associated with the aforementioned inflammaging pathologies such as IR (Cozma et al., 2018; Khoshdel and Eshtiaghi, 2019), nitro-oxidative stress (Bailey et al., 2013; Mozos and Luca, 2017), inflammation (Jain et al., 2014; Peyster et al., 2017) and reduced autophagy (Chen and Sun, 2019; McCarthy et al., 2019; Figure 3). In the following sections, we discuss stress-driven inflammaging as a mechanistic convergence between arterial stiffness and AD.

### Tissue-Specific Inflammation

The human body is constantly exposed to a multitude of noxious biological, chemical, and physical stimuli. Throughout evolution, the human body has developed mechanisms to recognize and to respond to those stimuli in order to maintain general health. Inflammation represents one of the body’s complex biological responses to those threats (Medzhitov, 2008). The concept of inflammation is generally divided into acute and chronic inflammation although both concepts overlap. Acute inflammation embodies the migration of immune cells to the site of injury facilitated by soluble immune-mediators (e.g., chemokines, acute-phase proteins, and cytokines). Depending on the severity of the injury, acute inflammation might not be sufficient to resolve the damage. Consequently, the prolonged and persistent exposure to stimuli evolves to a chronic inflammatory state in which tissue damage and fibrosis occurs (Germolec et al., 2018).

#### Neuroinflammation

For many decades, the brain was considered a fully immune-privileged organ, but this concept has faded and is replaced with a relative model of neuroinflammation involving a sophisticated immune response (Louveau et al., 2015). Despite a diversity in clinical symptoms, neurodegenerative disorders share common pathogenic cascades. Often the basis is the misfolding of proteins that aggregate in a disease-specific spatiotemporal pattern, which is associated with significant neuroinflammation contributing to disease onset and progression. This pathogenic pathway also appears pivotal in AD. Firstly, a strong link was established between AD and the innate immune response with microglial cells as its main players in the brain. Since microglia primarily locate in the vicinity of amyloid plaques (neuritic plaques) (Serrano-Pozo et al., 2011) and interact with Aβ and extracellular NFTs (Cras et al., 1991; El Khoury et al., 1998), AD-associated neuroinflammation was originally viewed as a passive response to these protein depositions. However, more recent insights grant an active contribution of inflammatory processes to AD pathogenesis, with the implication of both the innate and adaptive immune response, rendering neuroinflammation another AD hallmark (Heneka et al., 2015; Webers et al., 2020).

Microglia represent the main sensor of the brain’s innate immune system. Resting microglia display a ramified morphology and weak antigen presenting activity. Upon activation by tissue damage or pathogens, their morphology changes to an amoeboid-like shape allowing them to navigate through neuronal tissue. Local regeneration after neuronal damage is based on increased production of anti-inflammatory cytokines and neurotrophic factors, and the facilitation of phagocytosis of cell debris, as such promoting neuronal repair and survival (Kreutzberg, 1996; Nimmerjahn et al., 2005). In a healthy aging brain, microglia evolve toward a more inflammatory phenotype, as such contributing to the inflammaging phenomenon. This microglia priming or sensitization lies at the basis of a vicious cycle of exaggerated pro-inflammatory responsiveness significantly contributing to neurodegeneration in the AD brain (Norden and Godbout, 2013; Franceschi and Campisi, 2014).

Besides participating in several critical physiological functions, such as blood-brain barrier (BBB) integrity and the regulation of axonal outgrowth and myelination, astrocytes are important cellular regulators of the innate immune system. Reactive astrogliosis has also been demonstrated in the AD brain, especially in the vicinity of amyloid plaques (Medeiros and LaFerla, 2013). Astrocytes play a critical role in clearing Aβ plaques by the process of phagocytosis and secretion of Aβ-degrading proteases. Analogously to microglia, astrocytes release cytokines, interleukins, nitric oxide (NO), and other potentially cytotoxic molecules upon exposure to Aβ thereby exacerbating neuroinflammatory processes (Jensen et al., 2013; Skaper et al., 2018). Reciprocal interactions between microglia and astrocytes play an essential role in both healthy brain and neurodegenerative processes (Bouvier and Murai, 2015). Although both glial cell types appear to contribute to neuroinflammation in AD, the timing and mechanism of their involvement may differ substantially. The cellular characteristics of microglia support a stronger role in the initial stages of the neuroinflammatory cascade, while expression and release of tumor necrosis factor alpha (TNF-α) from those early recruited microglia could initiate a wave of signals complemented by the co-release of, for example, reactive oxygen species (ROS) to initiate reactive pathways in proximate astrocytes (Kreutzberg, 1996; Bouvier and Murai, 2015).

The involvement of the adaptive immune system in neurodegenerative disorders such as AD can also be presumed. Peripheral T lymphocyte activation and infiltration into cerebrospinal fluid and brain could be an important contributing factor to AD pathology. Infiltrated CD3+ T cells, mostly of the CD8+ subtype, were described in post-mortem human brain parenchyma (Itagaki et al., 1988; Rogers et al., 1988; Togo et al., 2002). In contrast to gliosis, the presence of CD3+ T cells often correlates stronger with tau- than with amyloid neuropathology (Merlini et al., 2018). Additionally, T-cell subsets in cerebrospinal fluid and peripheral blood show disease-related alterations. Decreased regulatory T cell, increased Th17 and increased CD8+ T cell levels were measured in blood of AD patients (Oberstein et al., 2018; Ciccocioppo et al., 2019; Burgaletto et al., 2020; Gate et al., 2020), while clonally expanded antigen-specific CD8+ T cells were present in cerebrospinal fluid of AD individuals (Gate et al., 2020), indicative of T cell trafficking into the central nervous system. In a similar fashion to the innate immune response, the adaptive immune system can develop a “friend or foe” relationship in AD. Various animal model-based studies have illustrated that controlled regulatory T cell depletion may be neuroprotective and mitigate AD progression via the suppression of harmful T cell subsets and even the modulation of the microglial response to amyloid-β deposition (Baruch et al., 2015; Dansokho et al., 2016; Baek et al., 2018; Mayne et al., 2020).

#### Vascular Inflammation

**FIGURE 4:**
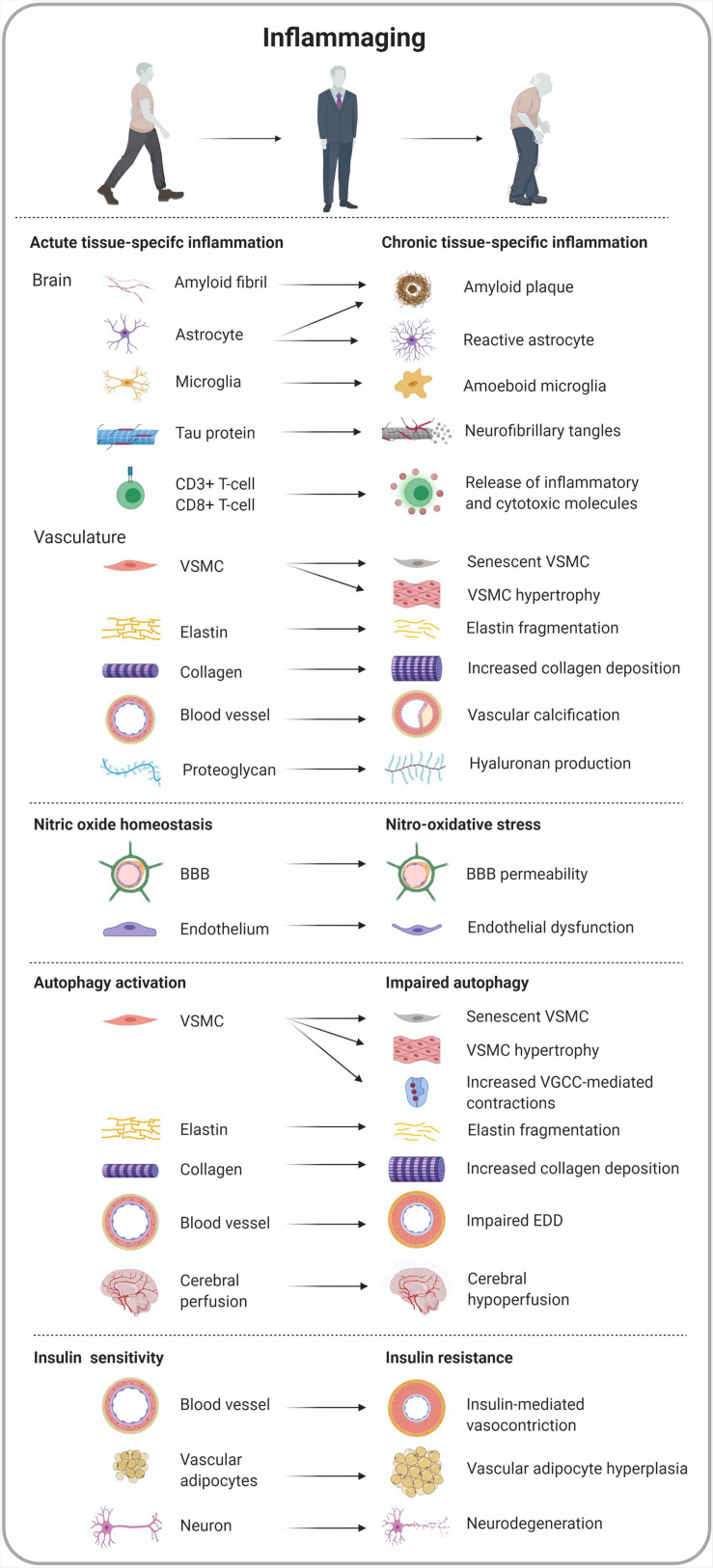
Overview of tissue-specific cell morphology and functionality in inflammaging-related conditions converging arterial stiffness and Alzheimer’s disease.

One key mechanism of arterial stiffness is aging of the arterial wall which is characterized by a chronic pro-inflammatory state coined “inflammaging.” Vascular inflammaging entails the production of proinflammatory stressors due to the failure of key molecular signaling cascades such as endothelin, renin/angiotensin II and the mineralocorticoid receptor signaling pathway (Lakatta, 2013; Wang et al., 2014). Consequently, the expression of pro-inflammatory transcription factors is facilitated (Wang et al., 2014). Vascular inflammation leads to structural remodeling of the arterial wall including vascular smooth muscle cell (VSMC) senescence and proliferation, vascular fibrosis, elastin breakdown, extracellular matrix (ECM) formation, and vascular calcification (Figure 4).

VSMCs are able to undergo several phenotypic switches causing proliferative, senescent and stiffened VSMCs to coexist in the aging arterial wall. Senescent VSMCs contribute to a pro-inflammatory state by the secretion of pro-inflammatory cytokines (IL-1, IL-6, and IL-17), monocyte chemoattractant protein-1 (MCP-1) and TNF-α (Csiszar et al., 2012; Accardi et al., 2016). It is hypothesized that this age-associated arterial secretory phenotype stimulates neighboring VSMCs to undergo a phenotypic switch in a juxtacrine fashion (Khan et al., 2010). Aged VSMCs in the arterial wall have an enhanced proliferation capacity marked by a greater percentage of cells in the S and G2/M phases and less cells in the G0/G1 phase. *In vivo* and *in vitro* research demonstrated the elevated expression of milk fat globule-EGF factor 8 protein (MFG-E8) and downstream integrin/ERK1/2 signaling to control VSMC proliferation and the cell cycle (Wang et al., 2012; Chiang et al., 2019). Moreover, old VSMCs exhibit an exaggerated migration/invasion capacity from the arterial media to the intima leading to age-associated diffuse intimal thickening (Spinetti et al., 2004; Fu et al., 2009; Wang et al., 2012). *In vitro* findings concluded the pivotal relay of MFG-E8 in VSMC migration and invasion (Fu et al., 2009). In addition, specific cleavage of the MFG-E8 protein leads to the formation of the amyloid protein, medin. This protein is deposited in the aortic media of the majority of European-Americans aged 50 and older (Wang et al., 2013; Migrino et al., 2017; Younger et al., 2020). Thus, the amyloidogenic MFG-E8/medin complex might accompany arterial stiffness with advanced age. Indeed, a positive correlation between PWV and serum MFG-E8 serum levels has been established in the elderly (Cheng et al., 2012).

A key feature of arterial aging is elastin fragmentation in the lamellae of the arterial medial layer. With time a greater strain is transferred to the less compliant collagen fibers in the ECM. Although long considered a passive phenomenon, elastin fragmentation can be acutely initiated by proteolytic elastase activity during instantaneous inflammatory responses. Certain matrix metalloproteinases (MMPs) are constitutively expressed by endothelial cells (ECs) and VSMCs upon inflammation, though these are counterbalanced by tissue inhibitor of metalloproteinases 2 (TIMP-2) in normal conditions (Park and Lakatta, 2012). Under the influence of inflammatory cytokines and the increased activity of cell adhesion molecules, macrophages, and neutrophils produce MMPs (including MMP-1, MMP-2, MMP-7, and MMP-9) (Galis and Khatri, 2002). These MMPs deteriorate the elastin-collagen crosslinking due to the degradation of basement membranes and their collagenolytic activity (Park and Lakatta, 2012), thereby stimulating uncoiled collagen, and thus arterial stiffening (Zieman et al., 2005). On a clinical level, the expression of relevant MMPs has been demonstrated to correlate to increased PWV levels (Wykretowicz et al., 2005; Yasmin et al., 2005; Vlachopoulos et al., 2007). A salient feature of ECM formation is enhanced collagen deposition. The complex meshwork of the ECM (collagen I, II, and III) is produced and maintained by VSMCs. The production of collagen molecules by VSMCs is governed by increased MMP-2 activated TGFβ1 signaling eventually leading to vascular fibrosis (Wang et al., 2006; Jiang et al., 2012). Furthermore, stiffer arteries comprise the ECM to resist compression. ECM inflammation causes an enhanced synthesis of glycosaminoglycans such as hyaluronan and altered proteoglycan structures. Hyaluronan forms a gel within the ECM by trapping water causing an impaired compression ability of the arterial wall (Maki-Petaja et al., 2012; Lorentzen et al., 2016; Maki-Petaja et al., 2016).

Similar to osteoblasts, VSMCs are able to undergo osteogenic differentiation when exposed to increased intracellular concentrations of calcium and/or inorganic phosphate (Neutel et al., 2020). Overexpression of calpain-1 reduces the calcification inhibitors such as osteopontin and osteonectin (Jiang et al., 2012; Tang et al., 2015). In addition, increased tissue transglutaminase (TG2) downregulates calcification-inhibitory genes (e.g., *Opn*) and upregulates calcification-promoting genes (e.g., *Runx2*) underlining its activation as an important molecular event in vascular calcification (Konoplyannikov and Nurminskaya, 2014; Chen W. R. et al., 2019).

#### Link Between Vascular Inflammation and Neuroinflammation

Aside the well-studied local inflammatory processes in arterial stiffness and AD, the effect of systemic inflammation on both pathologies has been put forward in recent years. A growing body of evidence suggests that systemic inflammation is associated or precedes arterial stiffness (Mahmud and Feely, 2005; Durham et al., 2018). Recently, it was reported that patients with chronic inflammatory diseases (e.g., rheumatoid arthritis, systemic sclerosis, systemic lupus erythematosus, and inflammatory bowel disease) present higher PWV values as compared to control subjects (Dregan, 2018; Vargas-Hitos et al., 2018). Moreover, at the level of the brain, systemic inflammation, marked by elevated circulatory proinflammatory cytokines, can shape a cerebral inflammatory milieu promoting neurodegeneration and AD (Paouri and Georgopoulos, 2019; Walker et al., 2019). Indeed, systemic inflammation has been demonstrated to induce cognitive decline and behavioral changes (Kahn et al., 2012; Anderson et al., 2015; Riazi et al., 2015; Sankowski et al., 2015). These findings have raised the question whether systemic inflammation plays a compensatory, mechanistic, or perhaps merely an associative role in the convergence between arterial stiffness and AD.

Accumulating experimental and clinical evidence suggests that Aβ-peptides, Aβ1–40 in particular, exert proinflammatory properties in the peripheral and cerebral vasculature (Noguchi-Shinohara et al., 2017; Stamatelopoulos et al., 2015, 2018; Visconte et al., 2018), making this an interesting biomarker. Several interventions using statins, angiotensin-converting enzyme inhibitors, angiotensin receptor blockers, angiotensin receptor/neprilysin inhibitors, β-blockers, diuretic agents, calcium-channel blockers, and hemodialysis showed the feasibility to manipulate the APP/Aβ turnover, aggregation of Aβ1–40 peptides or blockage of its inflammatory properties (Stakos et al., 2020). Furthermore, the age-associated amyloidogenic protein medin has been associated with vascular and cerebrovascular inflammation in recent years as it provokes endothelial dysfunction (Migrino et al., 2017; Degenhardt et al., 2020; Migrino et al., 2020; Younger et al., 2020). In this way, medin could also be considered an important inflammatory biomarker in the early-stages of the arterial stiffness and/or AD pathology. Recently it has been suggested that inflammaging increases the production of methylglyoxal, a dicarbonyl derivative of glucose. Increased levels of methylglyoxal may exacerbate stiffness and cognitive decline mainly in diabetic subjects (Dhananjayan et al., 2017).

Elucidating the underlying mechanisms of action of the aforementioned biomarker candidates might govern the crosstalk between systemic and local vascular and cerebral inflammation. On the long run, this understanding may provide the ground for new anti-inflammatory therapeutic approaches that successfully target arterial stiffness and/or AD.

### Nitro-Oxidative Stress

Although being the smallest gaseous signaling molecule, NO is responsible for maintaining homeostasis in a myriad of tissues and molecular pathways (Balaiya and Chalam, 2014) and embodies a key role in the pathogenesis of inflammation, though it is a double-edged sword (Sharma et al., 2007). In normal physiological conditions, NO exerts an anti-inflammatory function (Sarkate et al., 2017; Sherikar et al., 2019; Zhang et al., 2020a) while NO is also considered a pro-inflammatory mediator in pathological conditions, such as arterial stiffness (Sindler et al., 2011; Isabelle et al., 2012; Aroor et al., 2018; Chiba et al., 2019) and AD (Cifuentes et al., 2017; Tang et al., 2019; Austin and Katusic, 2020; Dubey et al., 2020; Stefano et al., 2020) mainly due to its contribution in nitro-oxidative stress (Pérez-Torres et al., 2020; Figure 4).

Vascular endothelial NO production is considered the most important vasodilator mechanism for the preservation of proper vasomotor function. The entire vascular system, from the heart to the smallest capillaries, is covered with an intimal endothelial monolayer which forms a barrier between circulating blood and surrounding tissues. By the release of NO, the vascular endothelium modulates basal and dynamic blood vessel diameter changes (Bauer and Sotníková, 2010). Indeed, the presence of arterial stiffness has previously been shown in rodent models of endothelial dysfunction by the inhibition of endothelial nitric oxide synthase (eNOS) with N(G)-Nitro-L-Arginine Methyl Ester (L-NAME) or by a genetic eNOS knock-out which causes hypertension and increased carotid-femoral PWV (Isabelle et al., 2012; Leloup et al., 2014). A diet-induced intervention in old mice with sodium-nitrite, on the other hand, led to de-stiffening of large arteries and normalized PWV values (Sindler et al., 2011).

The generation of endothelial NO and/or its bioavailability can be diminished by the increased generation of upstream oxidative stress. On itself, oxidative stress contributes to endothelial dysfunction by the rapid oxidative inactivation of NO through the excessive production of superoxide. As a result, eNOS activity uncouples and NO bioavailability decreases, creating a nitroso-redox imbalance and overall nitro-oxidative stress which precedes numerous vascular pathologies (Taverne et al., 2012; Sena et al., 2013). Excessive oxidative stress and consequent eNOS uncoupling have been demonstrated in mouse models (Xia et al., 2010; Herranz et al., 2012; Varadharaj et al., 2012; Yin et al., 2017) and patients with atherosclerosis (Varadharaj et al., 2012; Ismaeel et al., 2018) as well as in animal models (Adlam et al., 2007; Toral et al., 2018; Cheng et al., 2020; Dong et al., 2020) and patients with hypertension (Cengiz et al., 2015). Reactive oxygen species (ROS, e.g., superoxide) are formed, among other things, by multi-subunit NADPH oxidase (NOX) complexes from molecular oxygen using NADPH as electron donor. For many years, NOX expression was thought to occur only in infiltrating monocytes/macrophages and phagocytes. However, NOX expression has also been recently observed in vascular wall components (Panday et al., 2015). As such, NOX2 is considered the most prominent vascular NOX isoform as it is also expressed in VSMCs, adventitial fibroblasts, ECs, and perivascular adipocytes (Briones et al., 2011; Konior et al., 2014; Chen J. et al., 2017; Ling et al., 2018). A recent study demonstrated that the vascular infiltration of Nox2+ myeloid cells causes vascular inflammation resulting into endothelial dysfunction in a heart failure murine model ensuing myocardial infarction (Molitor et al., 2021). Furthermore, inhibition of the MRs/Nox2 redox signaling pathway via the downregulation of acid sphingomyelinase was shown to positively improve vascular adventitial remodeling in primary rat fibroblasts treated with angiotensin II (Li et al., 2019). In the cerebrovasculature of the AD brain, ROS are primarily generated via NOX2 in the disease-associated microglia. The enhanced activation of NOX2 in these microglia is associated with increased neuroinflammation and amyloid plaque deposition (Simpson and Oliver, 2020).

In this context, the link between arterial stiffness and AD has been mainly attributed to a loss of BBB integrity (Taheri et al., 2011; Wardlaw et al., 2013). Because the brain has no fuel reservoir, the brain draws energy from blood supplied by the cerebrovasculature through the BBB (Iadecola, 2017). The BBB serves as the first line of defense of the brain’s unique microenvironment against the systemic blood circulation (Sweeney et al., 2019). It has been proposed that downregulation of cerebrovascular eNOS expression predisposes certain brain areas to lose their BBB integrity (Soares et al., 2015; Al-Zaiti et al., 2018; Munji et al., 2019; Zhang et al., 2019; Bernstein et al., 2020). Intact endothelium and consequent preserved NO production in the cerebrovasculature is thus important to prevent stroke, cerebrovascular disease and neurodegeneration (Tabatabaei and Girouard, 2014; Khan et al., 2019; Haselden et al., 2020). A study from a metabolic point of view concluded that Type 1 diabetes driven eNOS-deficiency increases BBB permeability (Mayhan et al., 2015). Another study in a rat epilepsy model showed that increased cerebral eNOS expression led to BBB breakdown through provocation of a *status epilepticus*. This observation was further strengthened by the administration of an eNOS inhibitor (Ko et al., 2015). The opposite interpretation was derived from a study investigating the eNOS gene deletion in the BBB of a thiamine-deficient murine model. Here, knocking out eNOS restored BBB permeability, suggesting that eNOS-derived NO is an important factor in the cerebrovasculature of this pathology (Beauchesne et al., 2009). More specifically, an accumulative body of evidence speculates that the main culprit behind cerebrovascular dysfunction, as seen in AD, is oxidative stress and ROS production. It is known that ROS formation in the presence of Aβ peptides reduces NO bioavailability in the cerebrovasculature (Miller et al., 2010; Austin and Katusic, 2020). A rise of ROS seems to be mainly caused by Aβ-driven NOX2 activation eventually triggering neuroinflammation (Han et al., 2015; Wyssenbach et al., 2016; Hwang and Kim, 2018). This neuroinflammatory response involves the altered expression of tight-junctions in cerebrovascular ECs leading to a loss of BBB integrity and CAA (Carrano et al., 2011, 2012).

In addition to its role in the BBB, the importance of endothelial NO in the amyloidogenic processing of APP has been reported in the human and rodent cerebrovasculature (Austin et al., 2013; Austin and Katusic, 2016, 2020). A study in aged eNOS heterozygous mice demonstrated increased Aβ accumulation in cerebrovascular tissue without any increased amyloidogenic depositions in the brain (Austin and Katusic, 2020), likely due to only a partial loss of eNOS. However, a similar study in more severely aged eNOS knock-out mice evidenced increased APP expression and amyloidogenic processing in both vascular and brain tissue alongside microglial activation and impaired memory performance (Austin et al., 2013). Given its antiplatelet and vasorelaxant effects, reduced NO bioavailability, because of NOX2 induced oxidative stress (Malkov et al., 2020), likely promotes platelet hyperactivation and coagulation that worsens the pro-thromboembolic phenotype observed in the cerebral microvasculature of AD patients (Veitinger et al., 2014; Canobbio et al., 2015; Bose et al., 2019). Furthermore, it is known that platelets are a systemic source of APP processing and thus the generation of Aβ peptides (Chen M. et al., 1995; Foidl et al., 2020). Altogether, a decreased NO bioavailability in the cerebrovasculature not only drives cerebrovascular thrombosis, it also contributes to CAA which are both risk factors of stroke, cerebral hemorrhages and overall neuroinflammation.

To date, the exact role of nitro-oxidative stress in the linkage between arterial stiffness and AD is still under debate and needs further exploration in order to reach a more conclusive interpretation.

### Impaired Autophagy

Macroautophagy, hereafter referred to as autophagy, was described 50 years ago as a cellular homeostatic process that repurposes molecules for cell survival (De Duve and Wattiaux, 1966). However, in the last decades the role of autophagy has also been linked to physiological processes such as inflammation by its functionality in virtually all cell types and more particularly in immune cells (Figure 4). These observations not only underscored the importance of autophagy, but also sparked enthusiasm for targeting this cellular process in inflammatory diseases (Matsuzawa-Ishimoto et al., 2018). Autophagy maintains intracellular homeostasis by degrading unnecessary or dysfunctional cellular components in lysosomes. The process starts with the formation of double-membrane vacuoles or autophagosomes that engulf small portions of the cytosol such as protein aggregates, lipid droplets, and complete organelles. By fusing with a lysosome, the autophagosomes eventually turn into autolysosomes. During this final step, the cytoplasmic content of the autophagosomes will be degraded by lysosomal hydrolases. Growing *in vitro* and preclinical evidence indicates that basal autophagy in ECs and VSMCs is an essential process, mediating proper vascular function (De Meyer et al., 2015). Indeed, autophagy in vascular physiology plays a crucial role in lipid metabolism (Khawar et al., 2019), vascular reactivity (Michiels et al., 2015; De Munck et al., 2020b), homeostasis (Ouseph et al., 2015) and the maintenance of blood glucose and amino acid levels (Ezaki et al., 2011). Interestingly, autophagy is activated by stress-related signals, such as nutrient deprivation, oxidative injury, and endoplasmic reticulum stress. In this way, autophagy supports cell survival in unfavorable conditions. It also represents a reparative and life-sustaining process as autophagy induction extends life span in various species. However, aging is associated with a decline in autophagy in different tissues, including the arterial tree, due to a decreased expression of autophagy-related proteins (LaRocca et al., 2012), which in turn promotes arterial disease, such as arterial stiffness and accelerated atherogenesis. Indeed, *ex vivo* experiments with aortic segments isolated from mice with a VSMC autophagy defect (deletion of the essential autophagy gene Atg7) revealed attenuated compliance and higher arterial stiffness (De Munck et al., 2020c). Because the differences in compliance and stiffness are more pronounced when VSMCs are completely relaxed by the addition of exogenous NO, passive aortic wall remodeling, as shown by a decrease in elastin and an increase in collagen content, rather than differences in VSMC tone, is responsible for these effects (De Munck et al., 2020c). Passive remodeling of the aortic wall is supported by histological data showing an increase in medial wall thickness and elevated elastin fragmentation. Defective autophagy in VSMCs also leads to profound changes in Ca^2+^ homeostasis, resulting in higher basal Ca^2+^ stores, and larger voltage-gated calcium channel-mediated contractions (Michiels et al., 2015; De Munck et al., 2020b). In addition, autophagy deficiency in VSMCs triggers cellular hypertrophy and increases stress-induced premature cellular senescence (Grootaert et al., 2015). Because senescent cells lose their replication potential and have a pro-inflammatory secretory phenotype (i.e., Senescence Associated Secretory Phenotype or SASP), this condition has been linked to a number of age-related diseases, including atherosclerosis. Similar to defective autophagy in VSMCs, impaired autophagy in ECs has a major effect on vascular function. Decreased autophagy in ECs is associated with downregulation of eNOS and a reduction in arterial endothelium-dependent dilatation (EDD), indicating that autophagy preserves endothelial function by increasing NO bioavailability (LaRocca et al., 2012). Importantly, mounting evidence indicates that induction of autophagy could be a game-changer in the treatment of arterial stiffness and endothelial dysfunction (De Munck et al., 2020a). The natural autophagy enhancer spermidine, for example, restores NO-mediated EDD, normalizes arterial PWV and reduces blood pressure (LaRocca et al., 2013; Eisenberg et al., 2017). This outcome is associated with enhanced expression of autophagy markers in the arterial wall such as the autophagosomal marker LC3-II and the core autophagy machinery protein Atg3. Autophagy inducer trehalose elicits similar vascular- and cardioprotective effects (LaRocca et al., 2012).

Apart from the protective effects of autophagy against arterial stiffness and the interaction of the latter with AD, autophagy represents an important mechanism to clear toxic accumulation of misfolded proteins and dysfunctional organelles in the brain. It is worthwhile to mention that loss of the essential autophagy gene Atg5 causes neonatal lethality, yet Atg5-null neonates can survive if autophagy is restored in neurons (Yoshii et al., 2016), which confirms the major importance of autophagy in the brain. Consistent with this finding, impairment of autophagy is often associated with neurodegenerative disorders, including AD (Fujikake et al., 2018). Interestingly, Aβ1–40, which is the predominant component of cerebrovascular amyloid, inhibits proliferation of human brain vascular ECs through the induction of autophagy (Hayashi et al., 2009), and may explain the reduced vessel density and hypoperfusion of the hippocampus that characterize the initial stages of AD. This finding illustrates the complex role of autophagy (causative, protective or just a consequence of the disease) in AD (Liu and Li, 2019), and indicates that autophagy should be either induced or inhibited to prevent further development of AD, depending on the stage of the disease and type of target cells.

### Insulin Resistance

Insulin resistance is a pathological condition in which cells fail to respond to insulin so that the cellular uptake of glucose does not occur. Although IR is closely correlated to Type 2 diabetes mellitus (T2M) (Sampath Kumar et al., 2019), IR has also been established in non-diabetic individuals (Catena et al., 2015; Fu et al., 2017). In addition, IR is associated with cardiovascular morbidity and mortality (Ormazabal et al., 2018; Adeva-Andany et al., 2019; Markus et al., 2019). Although the underlying mechanisms explaining this relationship are not yet understood, arterial stiffness seems to be involved. Indeed, several observational and cross-sectional studies show a correlation between IR and arterial stiffness in (non-)diabetic persons with or without hypertension in different age groups, sometimes even prior to the development of glucose intolerance (Tounian et al., 2001; Seo et al., 2005; Agnoletti et al., 2013; Fang et al., 2014; Catena et al., 2015; Fu et al., 2017; Won et al., 2018; Markus et al., 2019). Moreover, obesity-driven IR has been shown to be an independent risk factor of diabetic vasculopathy and arterial stiffness (Jia and Sowers, 2014).

Impairment of vascular function and increased arterial stiffness by IR is linked to increased media, ECM and perivascular tissue thickness and endothelial dysfunction. In the medial layer of the arterial wall, insulin metabolic signaling normally leads to VSMC vasodilation meaning that IR leads to the impairment of vascular relaxation (Olver et al., 2019) and thus, endothelial function which is strengthened by both *in vitro* (Lee et al., 2012) and *in vivo* experiments (Doronzo et al., 2004; Lee et al., 2009). More specifically, the latter observations were accompanied by greater concentrations of ROS and impaired activation of NO signaling pathways (Padilla et al., 2015). Furthermore, stimulation of ECs and VSMCs by angiotensin and aldosterone leads to impaired insulin-mediated vasodilation through phosphorylation of insulin receptor substrate 1 (Cote et al., 2013). In addition, increased deposition and ECM remodeling have been reported in diet-induced obese conditions associated with IR (Kang et al., 2014; Williams et al., 2015). Outside of the arterial wall, most arteries comprise perivascular adipose tissue as a structural component and an abundant source of molecular paracrine function (Villacorta and Chang, 2015). In the context of IR, adipocyte hyperplasia occurs by the decreased expression of anti-inflammatory factors and the infiltration of pro-inflammatory immune cells in the perivascular adipose tissue (Aroor et al., 2013). As such, the Framingham Offspring and Third Generation cohorts illustrated a correlation between perivascular adipose tissue volume, aortic dimensions, and arterial stiffness (Thanassoulis et al., 2012; Figure 4).

Although the vasodilatory action of insulin is well-established, insulin can also constrict arteries via the autonomic nervous system. Already at modestly elevated insulin levels, an imbalance between the vasodilatory and sympatico-excitatory effects of insulin can occur (Gordin et al., 2019). One could argue that patients suffering from T2D display diminished vasoconstrictive effects because of advanced cardiovascular autonomic neuropathy (Braffett et al., 2020). It is therefore important to mention that the observed increased arterial stiffness measurements in individuals with T2D and IR might be underestimated because of a more pronounced vasodilatory effect of insulin.

As the most energy-demanding organ, the brain consumes up to 20% of glucose-derived energy in the body (Mergenthaler et al., 2013). Numerous devastating nervous conditions such as AD are characterized by neuronal energetic dyshomeostasis (Figure 4). To guarantee optimal energy management, the brain mainly obtains insulin from systemic circulating insulin although there is evidence that *de novo* insulin synthesis occurs in certain brain regions altogether making the brain an “insulin sensitive organ” (Banks et al., 2012). Whereas peripheral insulin predominantly acts as a metabolic regulator, the functionality of insulin in the central nervous system seems to resemble actions of the ancestral insulin protein, being a mitogenic growth factor and metabolic regulatory hormone. Banks and colleagues hypothesized that evolution of insulin’s functionality might have taken a divergent path in the periphery as compared to the central nervous system (Banks et al., 2012). The occurrence of IR in the brain was recently annotated as diabetes mellitus type III (T3DM) (Leszek et al., 2017; Candasamy et al., 2020). Several neurodegenerative features have been established as a consequence of systemic IR in AD, including metabolic and mitochondrial dysfunction (increased oxidative stress) (Wilkins et al., 2014), impaired systemic insulin signaling and miRNA deregulation (formation of NFTs) (Liu et al., 2014), neuroinflammation (activation of microglia and pro-inflammatory cytokines) (Gaspar et al., 2016; Chowdhury et al., 2018), impaired leptin signaling (loss of synaptic plasticity) (Arnold et al., 2018) and accelerated Aβ plaque formation (Petrov et al., 2015; Sallam et al., 2015). The interrelated link between AD, peripheral IR and T3DM has been established but remains poorly understood (Willette et al., 2015; Pugazhenthi et al., 2017; Denver et al., 2018). It is important to note that variable hormonal resistance syndromes exist and that these syndromes can occur independently from each other in different tissues.

To date, the relationship between IR, arterial stiffening and AD has been poorly studied. However, we recently found peripheral IR in an early-asymptomatic phase of the disease in young AD-relevant amyloidosis mice overexpressing the human APP with the Swedish double mutation [B6.Cg-Tg(Thy1-APP)3Somm/J, referred to as APP23]. This metabolic phenotype eventually evolved into a state of hyperinsulinemia at an older age. Throughout this longitudinal study, the animals showed no features of diabetes (Hendrickx et al., 2021). Previously, increased calorie intake was seen in addition to decreased body weights in the same single-transgenic AD mice, as well as in triple-transgenic AD mice where a hypermetabolic state was thought to be responsible (Vloeberghs et al., 2008; Knight et al., 2012). Recently, an overlap was found between hypermetabolism and brain atrophy in relation to obesity in healthy subjects (Pegueroles et al., 2019). In the context of arterial stiffness, we recently measured elevated PWV values in the above-mentioned APP23 mouse model after cardiovascular phenotyping in relatively young animals (Hendrickx et al., 2020a). In both studies (Hendrickx et al., 2020a; Hendrickx et al., 2021), animals demonstrated increased serum corticosterone levels at all ages compared to control littermates. In addition to what has previously been assumed (Vloeberghs et al., 2008; Knight et al., 2012), we hypothesize that the circulating stress hormone levels are the driving force for the development of hypermetabolism. The involvement of stress in the AD pathology has been extensively demonstrated and is now considered to be an important risk-factor of the disease (Bisht et al., 2018; Caruso et al., 2018; Dong and Csernansky, 2019). Hypermetabolism is known to be associated with IR (Willette et al., 2015; Gauglitz et al., 2008) and CVD (Clifton et al., 1981; Tulla et al., 1991). In this way, the mechanistic convergence of IR between arterial stiffness and AD could be explained as a cause of stress-driven hypermetabolism.

## Conclusion and Future Perspectives

Aging is a ubiquitous multifaceted biological process that represents a high-risk factor for many major disorders including CVD (e.g., arterial stiffness) and neurodegenerative disorders (e.g., AD). The process of aging is accompanied with increasing cortisol levels leading to chronic low-grade inflammation. Given their age-related nature, it is more than likely that the mechanistic convergence between arterial stiffness and AD is based on this inflammaging phenomenon. Apart from some lifestyle changes, no disease-modifying treatments are yet available for arterial stiffness and AD. From a future perspective, it is first of all important to consistently monitor features of inflammaging and to measure arterial stiffness early in clinical practice to diagnose and stop the progression of arterial stiffness and ultimately AD. Since a complex sequence of biological events seems to underlie the development of AD by arterial stiffness, it is very challenging to create an effective precision therapeutic treatment. Although precision medicine is still in its infancy, recent scientific efforts have already facilitated the elucidation of disease diagnosis and progression, as well as biomarker discovery through the combined use of “omics”-approaches and high-dimensionality data analysis (Hendrickx et al., 2020b). Finally, we argue that different forms of inflammaging bridge arterial stiffness and AD and that their early detection is important to timely intervene in the devastating pathological aging present in arterial stiffness and AD.

## Author Contributions

JH, WM, DV, and GD contributed to the writing and editing of this manuscript. All authors contributed to the article and approved the submitted version.

## Conflict of Interest

The authors declare that the research was conducted in the absence of any commercial or financial relationships that could be construed as a potential conflict of interest.
